# Echocardiographic Markers and Outcomes in End-Stage Liver Disease

**DOI:** 10.3390/jcm15072791

**Published:** 2026-04-07

**Authors:** Teodora Radu, Speranta Maria Iacob, Liliana Gheorghe

**Affiliations:** 1 Emergency Institute for Cardiovascular Diseases “Prof. Dr. C.C. Iliescu”, 022328 Bucharest, Romania; 2 “Carol Davila” University of Medicine and Pharmacy, 050474 Bucharest, Romania; msiacob@gmail.com (S.M.I.); drlgheorghe@gmail.com (L.G.); 3 Fundeni Clinical Institute, 022328 Bucharest, Romania

**Keywords:** liver cirrhosis, cirrhotic cardiomyopathy, left ventricle diastolic dysfunction, speckle tracking echocardiography, left ventricle global longitudinal strain, left atrial strain

## Abstract

**Background:** In end-stage liver disease (ESLD), cardiovascular changes are frequent and relate to the presence of hyperdynamic circulation. In 2019, diagnostic criteria for cirrhotic cardiomyopathy (CCM) were updated to include tissue Doppler and speckle tracking imaging in defining left ventricle (LV) systolic and diastolic dysfunction. Evaluation of diastolic function remains challenging, with frequent indeterminate cases and emerging evidence of worse prognosis. The aim of the present study was to evaluate the prevalence of LV systolic and diastolic dysfunction in cirrhosis, in correlation with liver disease severity and potential prognostic implications. **Methods:** We performed an observational, retrospective, non-randomized, single-center study that included 99 cirrhotic patients evaluated for liver transplant (LT) in a tertiary center. Liver disease severity and complications were analyzed with survival and echocardiography data to determine potential correlations with prognosis. For statistical analysis, IBM^®^ SPSS^®^ Statistics version 20 (Chicago, IL, USA) was utilized. A two-sided *p*-value < 0.05 was considered statistically significant. **Results:** Left atrial (LA) volume index (r = 0.230, *p* = 0.022), LA reservoir strain (r = 0.291, *p* = 0.003), and LA contraction strain absolute value (r = 0.223, *p* = 0.027) positively correlated with the severity of liver disease expressed by MELD Na score. LA dilation (≥34 mL/m^2^) was the most common echocardiographic finding. It was present in 69.7% of patients, with one third having severe LA dilation (>45 mL/m^2^), which was associated with worse survival (log rank *p* = 0.019). LA contraction strain with an absolute value higher than 16% was also associated with worse survival (log rank *p* = 0.024). In multivariable Cox analysis, only MELD-Na and LA volume index remained independently associated with mortality. Diastolic dysfunction appeared more prevalent among the non-surviving patients irrespective of the diagnostic criteria used (*p* = 0.023 for American Society of Echocardiography 2016 criteria; *p* = 0.032 for CCM 2019 criteria). On binomial logistic regression, the presence of significant diastolic dysfunction (>grade 1) was associated with an increased probability of composite end-point of death or LT in the presence of liver disease severity confounders. The use of the LA stiffness index in discerning diastolic function in patients with standard inconclusive evaluation may warrant further investigation. **Conclusions:** Echocardiographic alterations, particularly LA enlargement, are associated with liver disease severity and clinical outcomes in ESLD. These findings are hypothesis-generating and suggest a potential role for echocardiography in risk stratification, warranting validation in larger prospective studies.

## 1. Introduction

Cirrhosis is the final stage of chronic liver disease irrespective of the etiology. Cardiovascular changes in end-stage liver disease (ESLD) patients are common. They are related to the hemodynamic consequences of cirrhosis–hyperdynamic circulation, which is characterized by low systemic vascular resistances, high cardiac output, decreased systemic blood pressure, and compensatory activation of the sympathetic nervous system (SNS) and renin–angiotensin–aldosterone system (RAAS). This results in an increase in total body fluid, elevated heart rate, and central hypovolemia with excess fluid in the splanchnic circulation [1,2]. Due to reduced liver clearance, inflammatory cytokines and vasodilators enter systemic circulation [3]. Cardiac dysfunction can develop due to long-term sympathetic overstimulation causing myocyte damage. Excess angiotensin II promotes cardiac remodeling, proliferation, hypertrophy, and fibrosis. Certain cytokines and vascular mediators exhibit direct negative inotrope effects [4]. Cardiac dysfunction remains mostly asymptomatic as a result of the hemodynamic circumstances and can be easily missed. Yet it can evolve as decompensated heart failure (HF) in the presence of triggers like infection, transjugular intrahepatic portosystemic shunt (TIPS), or liver transplant (LT).

In 2005, at the World Congress of Gastroenterology in Montreal, the term “cirrhotic cardiomyopathy” (CCM) was defined for the first time as left ventricle (LV) systolic impairment or diastolic dysfunction in the presence of liver disease and in the absence of previous cardiac disease [5]. In time, the initial criteria became insufficient to evaluate LV function in the setting of evolving hemodynamic understanding and imaging progress.

In 2019, in agreement with the LV diastolic function guidelines published in 2016 by the American Society of Echocardiography (ASE) [6], the Cirrhotic Cardiomyopathy Consortium updated the diagnostic criteria by lowering the LVEF cut-off to ≤50% and including LV global longitudinal strain (LV GLS) < 18% to diagnose subclinical systolic dysfunction. Regarding the presence of diastolic dysfunction, three out of four markers for LV elevated filling pressure were required: septal e’ velocity < 7 cm/s, E/septal e’ ratio > 15, left atrial volume index (LAVi) > 34 mL/m^2^, and tricuspid regurgitation velocity > 2.8 m/s [7]. The reported prevalence of CCM was reduced from 50–70% (2005 criteria) to 29–55.7% (2019 criteria). Due to the use of speckle tracking, LV systolic dysfunction was more frequent using the 2019 criteria with a lower incidence of LV diastolic dysfunction [8,9].

Echocardiography remains the first-line investigation used to evaluate cardiac function in cirrhosis. Multiple parameters have been associated with prognosis, from heart chamber enlargement to diastolic dysfunction. The severity of cardiac involvement in cirrhosis was reported to correlate with the severity of the liver disease and was associated with worse outcomes after LT and TIPS [10].

Cirrhotic patients are reported to more frequently have LV hypertrophy and ascites patients more frequently have diastolic dysfunction [11]. One trial found a HF incidence after LT of 7% and that increased systolic pulmonary pressure (sPAP) was a risk factor [12]. In a large study reviewing the echocardiographic data obtained prior to liver transplant, the authors found that enlarged LA and high E/e’ ratio were related to early cardiovascular complications [13].

Previous studies have linked cardiac chamber dilation to cirrhosis severity and poor outcomes. Silvestre et al. reported that cirrhotic patients with a higher MELD score had significantly higher LA diameter and sPAP [14].

Diastolic dysfunction was associated with higher mortality in cirrhotic patients. In a recent study, the authors found diastolic dysfunction in 62.9% of patients using the ASE 2016 definition. Survival was shorter in patients with diastolic dysfunction and patients with E/e’ ratio >10 had an increased risk of death [15]. Another study showed that the severity of diastolic dysfunction correlated with the severity of liver disease, as the E/e’ ratio was higher and diastolic dysfunction was more frequent among patients with MELD higher than 15. Also the authors found that E/e’ > 10 was a predictor of mortality [16]. Cesari et al. demonstrated that enlarged LA and high E/e’ ratio were associated with mortality [17]. Other authors also showed a higher prevalence of diastolic dysfunction in Child C cirrhotic patients compared to Child A and B patients, with higher E/e’ ratio and LA volume index in the more severe group [18]. A recent study [19] demonstrated that echocardiographic 3D measured LA volume correlated with the severity of liver disease and was also associated with worse prognosis. Septal e’ < 7 cm/s was also correlated with adverse outcomes in cirrhotic patients after LT [20]. Ali and colleagues [21] found that 10% of cirrhotic patients developed HF after placing TIPS. They found that right atrial (RA) size, LV dimensions, and sPAP were correlated with the incidence of HF and that a 31 mmHg cut-off for sPAP could predict HF.

Despite all this data, diagnosing LV diastolic dysfunction on echocardiography remains challenging, with a 20% rate of inconclusive results in patients with heart failure with preserved ejection fraction (HFpEF) [22] and stress tests or invasive hemodynamic measurement would be necessary for further tailored diagnosis. Due to reduced effort tolerance and blunted chronotropic response to stress, use of effort/dobutamine stress echocardiography is difficult in cirrhosis. Guiding diuretic treatment using invasive hemodynamic measurements can be effective. Pelayo and colleagues found that in a group of cirrhotic patients with hepatorenal syndrome, 62% of patients had elevated pulmonary capillary wedge pressure (PCWP) or increased right atrial pressure, and that switching treatment from fluid supplementation to diuretic treatment in these patients improved prognosis [23]. Still, routine hemodynamic assessment in cirrhotic patients is difficult due to coagulopathy and associated bleeding risk.

In recent years, there has been continuous development of speckle tracking imaging, including defining age group normal cut-offs for left atrial strain, and further research in LA dysfunction. New diastolic function guidelines and strain use guidelines were published in 2025 [24,25]. The important role of LA strain in estimating LV filling pressure is highlighted. A step-wise approach to diagnosing diastolic dysfunction is proposed, using impaired e’ as an initial parameter. In the second phase, four parameters are included—average E/e’ > 14, LAVi > 34 mL/m^2^, LA reservoir strain ≤ 18, and E/A < 0.8 or >2. If e’ is impaired, only one parameter in step 2 is necessary for diagnosis. In the presence of normal e’, two additional parameters are required. The authors also proposed a new algorithm for diagnosing diastolic dysfunction and estimating LV filling pressure including the presence of either LA reservoir strain ≤ 18 or LAVi > 34 mL/m^2^ to help decide if a patient has grade 2 or 3 diastolic dysfunction when only one or two out of three parameters of elevated LV filling pressure are present (septal e’ < 6, E/e’ ratio > 15 or sPAP > 35 mmHg).

Left atrial stiffness, an invasive parameter measuring change in LA pressure relative to the change in LA volume, represents a fine marker of elevated LV filling pressures. It was adapted for non-invasive echo calculation using LA reservoir strain (LASr) to estimate myocardial stretch and E/e’ ratio to approximate filling pressures. Calculated using the formula (E/e’)/LASr, the left atrial stiffness index was associated with prognosis in HFpEF, an entity with common traits with CCM. A value above 0.26 correlated with increased risk of hospitalization and all-cause mortality [26].

Studies using speckle tracking imaging in cirrhotic patients have yielded divergent results so far regarding outcomes. Data referring to LA strain in cirrhosis is limited. Von Kochritz et al. reported a reduced mean absolute value of reservoir and conduit LA strain in patients with severe liver disease compared to controls [27]. Other authors did not demonstrate a correlation between LASr and the severity of cirrhosis, but reported an association with diastolic dysfunction severity [28]. Meucci and colleagues found that in cirrhotic patients receiving TIPS, a LASr below 35% and higher grades of diastolic dysfunction were associated with mortality [29].

LV GLS trial results in cirrhosis are also conflicting. Most initial studies reported lower absolute LV GLS values in cirrhotic patients compared to controls [30,31]. More recent trials reported different results. Some did not find differences between controls and cirrhotic patients [32] and others found increased LV GLS absolute values in ESLD patients [33]. Regarding the correlation of LV GLS with outcomes in cirrhotic patients, trials were both positive [34] and negative [29]. As was shown above, multiple echocardiographic parameters have been associated with prognosis in cirrhosis, but due to the use of different end-points, different cut-offs, and different definitions for heart failure or diastolic dysfunction, the results are not homogenous. The aim of our study was to evaluate the utility of various echocardiographic parameters, including LV GLS and LA strain, in assessing ESLD patients. Furthermore, the study investigated the prevalence of LV diastolic and systolic dysfunction using the 2019 CCM criteria in this population, focusing on their potential prognostic implications. The difficulty in evaluating LV filling pressures using current echocardiographic criteria was evident in the numerous indeterminate evaluations in the present study, as well as what has been previously reported by other authors. There is evidence of worse prognosis in these patients compared to those with normal diastolic function and, because of that, efforts to better stratify risk are justified. Considering the current trend to include LA strain as a marker of LA dysfunction and elevated LV filling pressure, we focused on the potential use of LA reservoir strain and LA stiffness index to discern diastolic function when the standard evaluation was inconclusive. We compared the results to a model of diastolic evaluation that used E/septal e’ ratio > 10 (a parameter that was previously linked to prognosis in ESLD) to differentiate undetermined cases. Given the retrospective, single-center design and limited number of events, this study was conceived as an exploratory, hypothesis-generating analysis rather than a definitive prognostic model.

## 2. Materials and Methods

We performed an observational, retrospective, non-randomized, single-center study that included a cohort of 99 patients with liver cirrhosis evaluated for LT candidacy in a tertiary center.

Liver disease etiology, severity, and related complications were analyzed together with survival and echocardiography data to determine potential correlations and prognostic implications.

Patients diagnosed with cirrhosis that were evaluated for LT in the gastroenterology ward during October 2023 and May 2025 were included. All participating patients signed their written informed consent. This study was performed in a single center and the local ethics committee approved it.

Patients under the age of 18 were excluded. Patients with poor echocardiographic window were excluded. Patients with previous cardiovascular disease were excluded (severe valve disease, heart failure with reduced ejection fraction, history of myocardial infarction (MI), significant coronary atherosclerosis with no prior revascularization, long standing atrial fibrillation). We did not exclude patients with cardiovascular risk factors, presence of paroxysmal atrial fibrillation, non-significant coronary atherosclerosis, or prior history of revascularization in the absence of MI.

Demographic data was collected including age, gender, height, weight, body mass index (BMI), and body surface area (BSA).

Medical history was recorded including cirrhosis etiology, severity (MELD and MELD Na scores), and complications (ascites, pleural effusion, encephalopathy, esophageal varices, variceal bleeding, thrombocytopenia, and hepatic cell carcinoma).

Cardiac evaluation consisted of relevant medical history, documenting cardiovascular risk factors (diabetes, obesity, smoking status, hypertension, dyslipidemia) and physical examination. ECG was routinely performed and corrected QT was noted using the Bazett formula.

Comprehensive transthoracic echocardiography was performed in all patients. As patients with an inadequate acoustic window were excluded, all patients had complete evaluations including speckle tracking imaging.

All echocardiographic examinations were performed by a single operator. Inter- and intra-observer variability were not assessed.

The GE Health Care VIVID E95 system was used. Standard 2D sections from parasternal long and short axes and apical 4, 5, 3, and 2 chamber view and subcostal view were obtained.

LA anteroposterior diameter, LV diameters, and wall thickness were measured from the parasternal long axis as recommended in ASE Chamber quantification guidelines [35]. Color Doppler, continuous Doppler (CW), and pulsed Doppler (PW) were used to assess mitral, aortic, tricuspid, and pulmonary valves. LA volume was measured in 4 chamber view focused on LA. Trans-mitral flow was evaluated using PW and early diastolic E wave and late atrial contraction A wave velocities, E/A ratio, and E wave deceleration time (DT) were registered. Tissue Doppler (TDI) was used to evaluate LV diastolic function; septal e’ and E/septal e’ ratio were noted.

Strain was measured using the software available on the echo machine. For LA strain (LAS) evaluation, dedicated apical 4 and 2 chamber views with focus on LA were obtained. LAS was measured using RR gating and reservoir (LASr); conduit (LAScd) and contraction (LASct) strain were noted. Absolute values were reported. All strain values were calculated as the average of 3 cardiac cycles. LA endocardial border tracing was corrected manually after initial semi-automated tracing and the region of interest was adjusted to the LA wall. The results were an average of the peak values of all segments generated by the software. A study published in 2023 [36] using only GE software reported cut-offs specific for age groups. In the case of the 50 to 60 years old group, the cut-offs were 34.3% for LASr, 18.5% for LAScd, and 16.3% for LASct. The LA stiffness index was calculated using the formula (E/septal e’)/LASr.

Dedicated sections for evaluating LV function using adjusted depth from standard apical 4, 2, and 3 chamber views were obtained. End diastolic and end systolic LV volumes (LVEDV, LVESV), stroke volume (SV), biplane Simpson LV ejection fraction (LVEF), and LV GLS were calculated using the echo dedicated software. The LV GLS was measured by averaging all segmental values in one section and next averaging the results from all 3 apical views. Tracing and region of interest were corrected manually after semiautomatic initial tracing. The values were represented as percentages and an absolute value lower than 18% was considered pathologic. LV systolic dysfunction was diagnosed based on the 2019 cirrhotic cardiomyopathy criteria (LVEF < 50% or LV GLS < 18%) [7].

Right atrial (RA) and right ventricle (RV) diameters and areas were measured from the 4 chamber view. Right ventricle function was assessed using fractional area change (RV FAC), the tricuspid annulus plane systolic excursion (TAPSE), and TDI RV free wall S wave velocity. sPAP was estimated using tricuspid regurgitation (TR) velocity to calculate RV/RA pressure gradient and the inferior vena cava (IVC) diameter and respiratory variation to estimate the pressure in the RA. For an IVC diameter ≤ 21 mm with respiratory variations of ≥50%, 5 mmHg were added to obtain sPAP.

LV diastolic dysfunction was defined in accordance with ASE 2016 diastolic evaluation guidelines [6] for patients with HFpEF:

- Grade 1 diastolic dysfunction—E/A ratio < 0.8.
- Grade 2 diastolic dysfunction—3 markers of elevated LV filling pressures present (septal e’ < 7 cm/s, E/septal e’ > 15, TR velocity > 2.8 m/s, LAVi > 34 mL/m^2^); if only 1 was present, the diastolic function was considered normal and if 2 were present, the diastolic function was considered indeterminate.
- Grade 3 diastolic dysfunction—E/A ratio > 2 and 2 factors of elevated LV filling pressures.

CCM diagnosis was made using the 2019 criteria established by the Cirrhotic Cardiomyopathy Consortium [7]. If only one marker of elevated LV filling pressures was present, it was considered that the patient had normal diastolic function and if 2 out of 4 were positive, the case was considered indeterminate.

In patients with indeterminate diastolic function, 2 models for assessing diastolic function were evaluated. One model tested the LA stiffness index—if above 0.3, patients were considered as grade 2 diastolic dysfunction or if the LA stiffness index was below 0.3, they were considered as normal diastolic function. The second model used E/septal e’ ratio—if patients had an E/septal e’ > 10, they were considered as grade 2 diastolic dysfunction; otherwise, no diastolic dysfunction was noted.

Blood test results were documented for cirrhotic patients (platelets, hemoglobin, leucocytes, CRP, creatinine, sodium).

Patients were followed up until 31 December 2025. No patient was lost to follow-up and all included patients had complete data sets. Date of evaluation and surviving status were noted for all patients and date of death and time until death were documented for the non-surviving patients. Only vital status and liver transplant status were assessed on follow-up. Date of liver transplant was noted. Vital status was assessed for LT recipients until the end of follow-up.

For statistical analysis, IBM^®^ SPSS^®^ Statistics version 20 (Chicago, IL, USA) was utilized. Continuous variables were depicted as the mean ± standard deviation for approximately normally distributed variables and as the median and range for non-normally distributed data. Categorical variables were summarized as absolute numbers and percentages. Correlations between scalar variables with normal distribution were performed with Pearson correlation coefficient. In the case of non-normal distribution, Kendell Tau’s non-parametric test was preferred because it is appropriate for ordinal or non-normally distributed continuous data. Chi-Square test was used to compare 2 nominal variables. For comparison between 2 groups, T test was used. A two-sided *p*-value < 0.05 was considered statistically significant. Considering the high number of statistical tests performed on a relatively small data set, a significant risk of type 1 error existed and in accordance, in certain cases, only a *p* < 0.01 was reported.

Survival probabilities were estimated using the Kaplan–Meier method, and differences between groups were compared using the log-rank test. The end-point was death. During follow-up, 29 patients died. The time period was defined between cardiac evaluation and death. Patients who survived by the end of the study follow-up were censored, with the survival analysis including only non-surviving patients. A *p*-value of <0.05 was considered statistically significant.

A Cox proportional hazards model was employed to determine the association between the covariates MELD Na score, LA volume index, and LA contraction strain and mortality end-point, adjusting for potential confounders. Due to the small number of events, only 3 covariates were tested. Proportional hazard assumption was verified using partial residuals plots. Hazard ratios (HRs) and their 95% confidence intervals (CIs) were reported.

Binomial logistic regression was used to evaluate the effect of diastolic dysfunction (grades 2 and 3 and indeterminate cases) on composite end-point of death or liver transplant (45 events in total). The model was adjusted for the cofactors MELD Na score and pleural effusion. Linearity assumption was tested using the Box–Tidwell procedure. We assessed multicollinearity using the variance inflation factor (VIF < 5) and tolerance (>0.1). The goodness of fit of the model was assessed using the Hosmer–Lemeshow test (*p* > 0.05). Results are presented as odds ratios (ORs) with 95% CI.

## 3. Results

The baseline demographic, liver disease, and cardiovascular characteristics of the studied cirrhotic cohort are presented in Table 1.

The mean age was 53. Male gender was predominant. The most prevalent liver disease etiology was alcohol followed by chronic viral hepatitis. The mean MELD Na score was 15.73—corresponding to a 90 day mortality risk of 6%. The majority of patients had a history of ascites, thrombocytopenia, and esophageal varices. During follow-up, 29 patients died and 17 underwent liver transplant.

Regarding cardiovascular risk, the most common risk factors were smoking history, hypertension, and obesity. Six patients had paroxysmal atrial fibrillation and 11 patients had a personal history of coronary artery disease (CAD)—non-significant coronary lesions or prior elective revascularization. A cirrhosis-dedicated cardiovascular risk score was calculated in all patients—mCAD LT. The mean value placed the population at moderate risk for CAD.

The mean values of the echocardiographic parameters are presented in Table 2.

The majority of parameters are within normal reported range, with the exception of LA dimensions, which are enlarged, and the LA strain values, which are lower in absolute value than predicted for age group [36].

Regarding the most frequent pathological echocardiographic findings in the cirrhosis cohort, data is presented in Table 3.

The most common abnormality encountered in the cirrhotic population was LA enlargement, with a third of patients having severe LA dilation (LAVi > 45 mL/m^2^). The presence of diastolic dysfunction and LV hypertrophy was also common.

Regarding the classification of diastolic dysfunction, most patients had grade 1 diastolic dysfunction. Only seven patients had grade 2 and 3 diastolic dysfunction and 14 patients had an inconclusive evaluation.

In accordance with the higher incidence of diastolic dysfunction, TDI septal e’ < 7 cm/s and E/septal e’ > 10 were frequent among ESLD patients.

LVEF < 50% was present in only two patients, but when LV GLS was considered, an absolute value equal or below 18% was noticed in 18 patients.

CCM diagnosis varied from 60.6% when using the 2005 diagnostic criteria to 19.2% when the 2019 diagnostic criteria was used. LV systolic function was present in only 10.1% of patients when the 2005 criteria were used, compared to 19.2% when utilizing the 2019 criteria. As seen in Table 4, this is mainly driven by introducing LV GLS in the diagnostic criteria. Inversely, when diastolic dysfunction is concerned, using the 2019 criteria reduces the diagnosis from 56.6% to just 6.1% with 14 indeterminate cases.

Some echocardiographic parameters varied significantly with MELD Na score. All parameters had positive correlations—higher values were associated with higher MELD Na scores, suggesting a possible correlation with the severity of liver disease. Data is presented in Table 5.

Comparing the echocardiographic data between patients with a MELD Na higher than 15 and those with less severe disease, more parameters had statistically significant differences between the two groups. The higher values were consistently present in patients with higher MELD Na scores, supporting a probable connection between severity of cirrhosis and cardiac changes. LA dimensions were increased in patients with more severe liver disease, but LA strain absolute values were higher in this group. Data is available in Table 6. To account for potential type 1 error, only results with *p* ≤ 0.01 were reported.

We analyzed liver disease parameters and echocardiographic data in relation to the time until death for the non-surviving patients and the data is presented in Table 7.

Liver disease severity parameters (MELD-Na and MELD scores) inversely correlated with survival time among non-survivors. The presence of pleural effusion was associated with a shorter survival time. Regarding echocardiographic parameters, severe LA dilation seemed to be associated with shorter survival. LASct was inversely correlated with survival time, and an absolute value lower than 16% appeared to be associated with longer survival, although this association did not persist in multivariable analysis.

**Figure 1:**
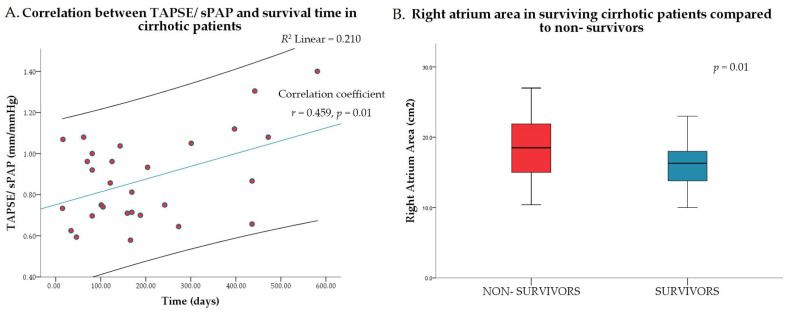
(**A**) Correlation between TAPSE/sPAP and survival time in cirrhotic patients. (**B**) Right atrium area in surviving patients compared to non-survivors.

Pulmonary systolic pressure correlated negatively with survival time. RV systolic function parameter TAPSE/sPAP positively correlated with survival time (Figure 1A). Higher RAA values were more common among the non-surviving patients (*p* = 0.01 Figure 1B). Other parameters evaluating RV function like FAC, TDI RV free wall S, or TAPSE did not correlate with survival time and did not differ among surviving and deceased patients.

**Figure 2:**
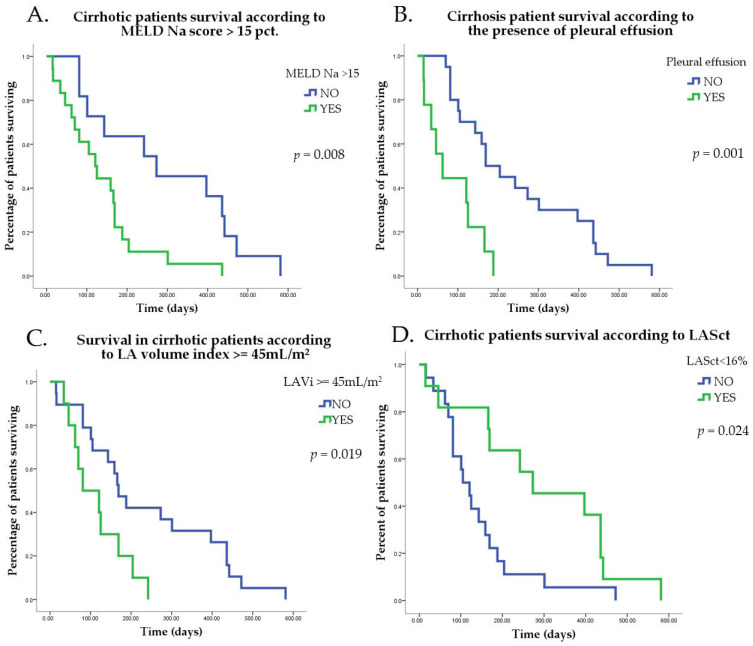
(**A**) Kaplan–Meier curve comparing survival in patients with Meld Na > 15 and those with less severe liver disease. (**B**) Kaplan–Meier curve comparing survival in patients with pleural effusion and those without. (**C**) Kaplan–Meier curve comparing survival in patients with severe LA dilation—LAVi > 45 mL/m^2^ and those with less dilated LA. (**D**) Kaplan–Meier curve comparing survival in patients with LA contraction strain absolute value lower than 16 and those with higher values.

Kaplan–Meier survival curves for MELD Na, pleural effusion, LA volume index > 45 mL/m^2^, and LASct < 16% are presented in Figure 2A–D.

Multivariable Cox proportional hazards analysis (Table 8) showed that, after adjusting for MELD-Na, the LA volume index (*p* = 0.031, HR 1.045, 95% CI 1.004–1.087) remained independently associated with mortality, whereas LA contraction strain did not.

**Figure 3:**
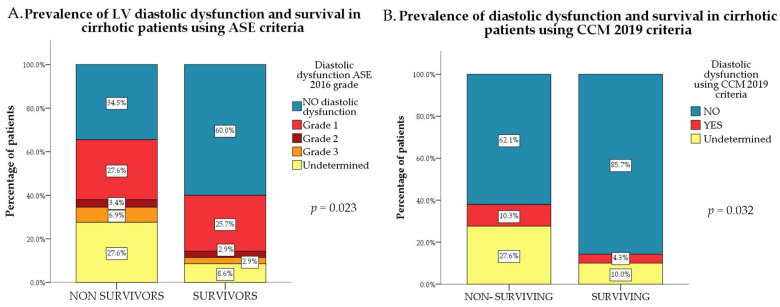
(**A**) Prevalence of LV diastolic dysfunction according to ASE 2016 criteria in surviving patients compared to non-survivors. (**B**) Prevalence of LV diastolic dysfunction according to CCM 2019 criteria in surviving patients compared to non-survivors.

The presence of diastolic dysfunction, irrespective of the criteria used, was more frequent among non-survivors (*p* = 0.023, Figure 3A for ASE 2016 criteria; *p* = 0.032, Figure 3B for CCM 2019 criteria and *p* = 0.019).

Binomial logistic regression (Table 9) was used to evaluate the effect of diastolic dysfunction (grades 2 and 3 and indeterminate cases) on the composite end-point of death or LT (45 events in total). The model was adjusted for MELD-Na score and pleural effusion. Linearity assumption was tested using the Box–Tidwell procedure. We assessed multicollinearity using the variance inflation factor (VIF < 5) and tolerance (>0.1). The goodness of fit of the model was assessed using the Hosmer–Lemeshow test (*p* > 0.05). The model was statistically significant (*χ*^2^(3) = 25.432, *p* < 0.0005), indicating that it distinguished between outcomes. The model explained 30.3% (Nagelkerke R^2^ 0.303) of the variance of the composite end-point and correctly classified 71.7% of cases. Patients with significant diastolic dysfunction (grades 2 and 3 and indeterminate cases) were 4.9 times more likely to experience the composite end-point compared to patients with no or mild (grade 1) diastolic dysfunction (*p* = 0.009, OR 4.876, 95% CI 1.481–16.058).

Concerning other speckle tracking imaging parameters, LV GLS (either continuous absolute value *p* = 0.33, or categorical variable higher than 22% *p* = 0.13 or lower than 18% *p* = 0.43), LASr (either continuous value *p* = 0.89, or categorical variable lower than 24% *p* = 0.56 or lower than 33% *p* = 0.42), and LAScd (either continuous absolute value *p* = 0.94 or categorical variable lower than 16% *p* = 0.46) were not associated with survival.

Regarding other diastolic dysfunction parameters, we did not demonstrate a correlation between survival status and septal e’ value below 7 (*p* = 0.2), E/septal e’ > 15 (only 3 patients, *p* = 0.65), or E/septal e’ ratio > 10 (*p* = 0.28).

CCM diagnosis was not associated with mortality (*p* = 0.059) or with survival time (*p* = 0.054) in the studied population.

We investigated the potential role of LASr, LA stiffness index, and E/septal e’ in determining diastolic function in patients with an inconclusive standard evaluation.

We used a cut-off value of LASr lower than 24% to establish indeterminate diastolic function cases as grade 2 diastolic dysfunction. Out of 14 indeterminate cases, two patients were distributed to the grade 2 diastolic dysfunction group based on LASr < 24% and 12 to the normal diastolic function group. Diastolic dysfunction prevalence analyzed in this situation was more frequent among non-survivors (13.8% versus 5.1%), but it did not reach statistical significance (*p* = 0.17).

The LA stiffness index was not correlated with MELD Na score. It was associated with age (Kendell Tau’s r = 0.362 *p* < 0.001) and with LV GLS (Kendell Tau’s r = 0.277 *p* < 0.001). The LA stiffness index did not correlate with survival.

We analyzed patients with indeterminate diastolic function, confirming diastolic dysfunction if the LA stiffness index was above 0.3. This analysis rendered a total of 31 cases of CCM and 15 diastolic dysfunction cases.

**Figure 4:**
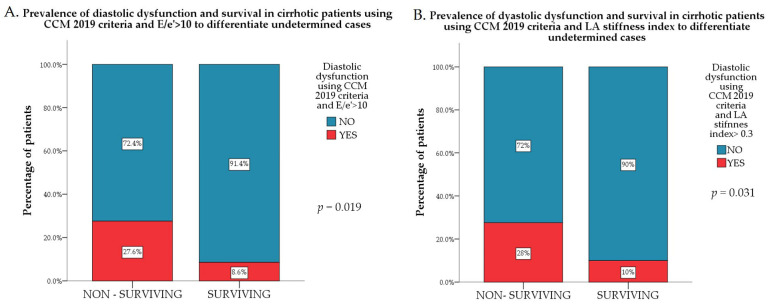
(**A**) Prevalence of LV diastolic dysfunction according to CCM 2019 criteria using E/e’ > 10 to differentiate indeterminate cases in surviving patients compared to non-survivors. (**B**) Prevalence of LV diastolic dysfunction according to CCM 2019 criteria using LA stiffness index > 0.3 to differentiate indeterminate cases in surviving patients compared to non-survivors.

We also analyzed inconclusive diastolic dysfunction cases using E/septal e’ ratio > 10 as cut-off for establishing the presence or absence of diastolic dysfunction. This resulted in 29 CCM cases and 13 patients with diastolic dysfunction. Diastolic dysfunction remained more prevalent among non-survivors (*p* = 0.019; Figure 4A) using E/septal e’ > 10 in a similar way as when using the LA stiffness index (*p* = 0.032; Figure 4B).

## 4. Discussion

Liver disease severity has been linked to a magnitude of cardiac changes in cirrhotic patients in multiple previous studies. The physiopathology relies on the presence of hyperdynamic circulation, a hallmark of cirrhosis. Splanchnic vasoactive substances (carbon monoxide, nitric oxide) reach systemic circulation, causing a reduction in vascular resistances and mean arterial pressure, activating SNS and RAAS. This leads to an increase in circulating catecholamine levels, elevated heart rate, and sodium and water retention, resulting in a persistent state of high cardiac output at rest, with increased preload and reduced afterload. The physiologic interplay between intrathoracic pressure respiratory variations and the response of the right and left ventricles to preload and afterload variations were recently summarized by Di Cristo et al. [37]. Diastolic dysfunction is more evident in patients with ascites due to diaphragm ascension, increased intrathoracic pressure, and volume overload. Myocardial fibrosis and hypertrophy induced by RAAS activation also contribute to aggravate diastolic dysfunction [30]. The value of hemodynamic changes in predicting outcome in cirrhosis was demonstrated by Turco et al. [38] in a trial evaluating hepatic and right-heart catheterization. As a result of atrial and ventricular strain, E/e’ and TAPSE/sPAP are highly load-dependent in ESLD; their association with more advanced cirrhosis and pulmonary congestion likely reflects the hyperdynamic, volume-overloaded hemodynamic state rather than purely intrinsic myocardial mechanics, which complicates their interpretation as prognostic markers.

In the present study, we demonstrated a possible connection between liver disease severity expressed by MELD Na score and echocardiographic changes. Patients with MELD Na higher than 15 had larger LA and LV dimensions and higher E wave velocity. The LV systolic function assessed by LV GLS and LVOT VTI appeared increased and pulmonary artery systolic pressure and RV systolic function parameters (TAPSE, TDI S) were also elevated. Cardiac output and stroke volume, right heart chamber dimensions, and speckle tracking imaging parameters correlated positively with MELD Na. This is in accordance with other trials, as larger LA dimensions, higher sPAP, higher E wave velocity, higher E/e’ ratio, and increased LV GLS and LVEF were observed in patients with more severe liver disease [14,18,28,39].

As previously discussed, many trials report LV diastolic dysfunction-related echocardiographic parameters as significant in regard to liver disease progression and prognosis [15,16,18]. In our study, the presence of diastolic dysfunction appeared to be more frequent among patients with negative outcome irrespective of the diagnostic criteria used. Using binomial multivariate regression, we demonstrated that diastolic dysfunction, defined as more severe than grade 1, maintained an effect on the composite end-point of death or LT in the presence of MELD Na and pleural effusion.

Although E/septal e’ ratio > 10 and septal e’ < 7 cm/s were previously linked to negative outcome, we could not find an association with prognosis in the present study. Despite that, we identified a high prevalence of LA enlargement in the studied population. In the absence of congenital heart disease or primary mitral valve disease, LA dilates in relation to LV structural changes, diastolic dysfunction, and increase in LV filling pressure. A recent study identified LA dilation as a significant predictor of mortality in HFpEF patients. In the presence of normal LV geometry, LA dilation determined a 28% increase in mortality risk [40]. In cirrhosis, there is evidence of LV hypertrophy, diffuse myocardial fibrosis, and increase in extracellular volume documented by cardiac magnetic resonance (CMR) relating to poor outcomes [41]. Progression of LA enlargement in cirrhotic patients has been linked to the hemodynamic changes induced by chronic liver disease [14,17,39]. A correlation between mortality and LA dilation was observed in published trials [17,19,28]. In the present study, we found an association between severe LA dilation and negative outcome on the Kaplan–Meier survival analysis (log-rank *p* = 0.019). The LA volume index remained associated with mortality in the presence of MELD-Na in multivariable Cox proportional hazards analysis, suggesting that chronic LA remodeling may carry prognostic information beyond liver disease severity alone.

We also identified a possible correlation between MELD Na and left atrial strain—higher strain values were present in patients with more severe liver disease. A different article reported a similar trend between MELD score and atrial strain, with an inverse correlation between outcome and LASr [42]. This finding can be associated with the dependence of strain parameters on loading conditions [43], since patients with more severe cirrhosis have more important hemodynamic alterations, volume overload, and higher cardiac output, thus making strain values difficult to interpret. Other authors found that a lower LASr than 35% was associated with negative prognosis [29]. In the present study, patients with lower LASct absolute values appeared to have a better outcome on Kaplan–Meier analysis (log rank *p* = 0.024), but the parameter did not maintain its effect in the presence of MELD Na and the LA volume index when multivariate Cox proportional hazard analysis was performed. Further trials to better understand the role of atrial strain in the specific setting of cirrhosis are warranted.

The clinical implications of LV diastolic dysfunction and LA dilation in cirrhotic patients can be unmasked in certain scenarios like active infection, hepatorenal syndrome, liver transplant surgery, and TIPS. In the context of sepsis, due to endotoxins and inflammatory cytokines with negative inotrope and vasodilatory effect, patients with previous subclinical cardiac impairment cannot maintain cardiac output, resulting in reduced renal perfusion, renal injury, and hemodynamic compromise. Tailoring treatment according to invasive hemodynamic measurement has been proven effective, demonstrating that a significant amount of these patients have high right atrial pressure and elevated PCWP, thus showing signs of cardiac impairment [10,23]. Liver transplant surgery is associated with abrupt hemodynamic shifts during the time of the operation. After transplant, the hemodynamic effects of cirrhosis reverse with the increase in peripheral vascular resistances, translating into an increase in LV afterload. One trial found that half of the LT recipients had radiologic signs of pulmonary congestion in the intensive care unit (ICU) after LT surgery [44]. In the context of the sudden afterload increase and previous LV subclinical dysfunction, cardiac output is reduced with consecutive end diastolic LV pressure, LA pressure, PCWP, and pulmonary pressure increase [45]. HF-related mortality after LT was reported as high as 15% [30]. Diastolic dysfunction parameters—reduced septal e’ and elevated E/e’—correlated with early cardiac events after LT [13,20]. HF after LT can also determine longer ICU stay, higher infection risk, and graft failure. TIPS can also have a profound hemodynamic effect, resulting in increased RV preload and high cardiac output, with 10% of patients developing HF [21] and some patients evolving with persistent diastolic dysfunction and worse outcome [16,46]. Atrial fibrillation and atrial flutter are common among cirrhotic patients and also after LT. They are related to atrial enlargement and myocardial fibrosis and also with liver disease severity [47].

Assessing LV filling pressures using echocardiography can be associated with a high number of indeterminate cases, as was the case in the present study (14.1%). In the context of an evident correlation between diastolic dysfunction and negative prognosis, efforts should be made to better characterize LV diastolic function.

In some cases, diastolic dysfunction is not evident on resting conditions. In healthy individuals, the E/e’ ratio does not increase during physical exercise, whereas in patients with diastolic dysfunction, there is a sharp increase in E wave not paralleled by e’. Invasive studies have shown that end diastolic LV pressure increases during effort in HFpEF patients, followed by an elevation in pulmonary pressure [48]. Reduced effort tolerance, blunted chronotropic response, and coagulation disorders make effort echocardiography and invasive hemodynamic studies difficult in cirrhosis.

One trial found that 21% of HFpEF patients had indeterminate diastolic function and demonstrated a higher risk of heart failure, atrial fibrillation, and stroke compared to normal diastolic function individuals. The authors used a LASr cut-off of 24% to stratify risk [49]. In the recent update on diastolic function assessment in HFpEF [25], LASr was introduced to better assess LA filling pressures and diastolic dysfunction grade with LASr ≤ 18 indicating high filling LV pressure. Also the authors proposed a step-wise approach algorithm.

In the studied population, a very limited number of patients had LASr ≤ 18% or E/septal e’ > 15, thus entailing little consecutive value in establishing diastolic function. When using LASr < 24% to discern indeterminate diastolic function cases, the result was not statistically significant. An LA stiffness index above 0.3 differentiated patients with indeterminate diastolic function in a similar way compared to adjusting E/septal e’ to a cut-off of 10. In both scenarios, prevalence of diastolic dysfunction seemed to remain higher among non-surviving patients. We used E/septal e’ > 10 to compare LA stiffness results due to the fact that multiple cirrhosis trials have reported it as a determinant of outcome. Of course, the present study can only generate a hypothesis. Evolution of imaging techniques and changes in diastolic evaluation algorithm recently proposed for heart failure patients warrant future research in cirrhosis.

The main limitations of the study are its single center and retrospective nature. Furthermore, the echocardiography data was recorded by a single operator, with no data available regarding inter-observer and intra-observer variation. The patient cohort was relatively small and in this context, the high number of statistical tests performed introduced a risk of type 1 error. In some cases, we only reported results with a *p* ≤ 0.01 but isolated remaining *p*-values close to 0.05 requires a cautious interpretation. Since the study was performed in a tertiary center, the cirrhotic patients were mostly referred from other centers and this implied a preselection. In this context, the study findings might not be representative for cirrhotic patients in general. Even excluding patients with known significant cardiac pathology, the age and prevalence of cardiovascular risk factors among patients on the LT waiting list is increasing and this can also be remarked in the present study cohort. The relatively high prevalence of hypertension, obesity, and diabetes introduced further confounding effects that could not be accounted for due to the limited number of events. The study did not include invasive catheterization measurements to compare echocardiographic data. The follow-up period was variable and short in some of the cases. This influenced the number of events registered during follow-up (only 29 deaths and 17 patients who underwent LT). In consequence, Kaplan–Meier analysis included a small number of cases. Furthermore, due to the low number of events, Cox multivariate survival model could not include more than three covariates, limiting the possibility to adjust for more confounders than Meld Na. We did not investigate for the presence of heart failure or echocardiographic changes occurring after LT. Also the present study did not include patients with TIPS, which is a major trigger for HF. A comparison between patients with and without HF after the TIPS or after LT would have better exposed echocardiographic parameters with potential prognostic value. Including cardiac biomarkers (NT pro BNP and hsTnI) would have resulted in valuable information but they were not systematically available. LT introduced a confounding effect by increasing survival and reducing the number of events.

Because liver transplantation was handled as a censoring event and post-transplant outcomes were not analyzed, our approach does not account for competing risks and may underestimate or overestimate the association between echocardiographic parameters and mortality. The limited number of deaths and composite events also restricted the number of covariates that could be included in multivariable models, increasing the likelihood of residual confounding.

Given the retrospective, single-center design, limited sample size, and the large number of echocardiographic parameters explored, our analyses are inherently exploratory and susceptible to type I error. Isolated *p*-values close to 0.05, especially for subgroup or threshold-based findings, should therefore be interpreted with caution and viewed as hypothesis-generating.

## 5. Conclusions

Echocardiographic changes in cirrhotic patients correlated with liver disease severity. Severe LA dilation was associated with worse prognosis and remained associated with mortality after adjustment for MELD-Na, whereas lower absolute LA strain values did not. Strain values in more severe ESLD appear highly sensitive to loading conditions and should be interpreted in the context of the hyperdynamic circulation; specific cut-offs for cirrhotic patients obtained with comparative invasive hemodynamic studies would improve their clinical utility. In contrast, LA dilation represents a marker of chronic cardiac structural change and diastolic dysfunction induced by cirrhosis physiopathology. Evolution of imaging techniques and recent changes in diastolic evaluation algorithms proposed for heart failure patients warrant further research in cirrhosis, and the LA stiffness index may represent a promising non-invasive tool, but its role in ESLD requires confirmation in larger, prospective, and ideally multicenter studies with invasive hemodynamic and outcome validation.

## Figures and Tables

**Table 1:** End-stage liver disease patients’ baseline characteristics (N = 99).

ESLD Patients’ Baseline Characteristics			
Age	53.57 (±11.11)	Grade 3	27 (27.3%)
Gender (M)	66 (66.6%)	Thrombocytopenia (<100.000 platelets/µL)	56 (56.6%)
Weigh (kg)t	80.26 (±17.44)	Pleural effusion history	21 (21.2%)
Height(m)	1.73 (±0.09)	Portal vein thrombosis	16 (16.2%)
Body surface area (BSA)	1.95 (±0.24)	Esophageal varices	77 (77.8%)
Body mass index (BMI)	26.81 (±5.1)	Esophageal varices grade	
Liver disease etiology		Grade 1	31 (31.3%)
Viral	38 (38.4%)	Grade 2	20 (20.2%)
VHB	5	Grade 3	22 (22.2%)
VHB + VHD	17	Grade 4	3 (3%)
VHC	13	Hepatocarcinoma	35 (35.4%)
VHC + VHB + VHD	3	Death	29 (29.3%)
Non-viral	61 (61.6%)	Liver transplant	17 (17.2%)
Alcohol	39 (39.4%)	Cardiovascular characteristics	
Other	22 (22.2%)	Hypertension	36 (36.4%)
Liver disease severity		Smoking history	45 (45.5%)
MELD	14.6 (±6.1)	Diabetes type 2	29 (29.3%)
MELD-Na	15.73 (±6.8)	Obesity	31 (31.3%)
Gastrointestinal bleeding history	24 (24.2%)	Dislipidemia	22 (22.2%)
Encephalopathy history	21 (21.2%)	Atrial fibrillation (paroxysmal)	6 (6.1%)
Ascites history	56 (56.6%)	CAD personal history	11 (11.1%)
Ascites grade		Cardiovascular disease family history	9 (9.1%)
Grade 1	15 (15.2%)	mCAD LT	7.48 (±3.39)
Grade 2	14 (14.1%)	QTc (ms)	433.99 (±29.35)

**Table 2:** Echocardiographic parameters in cirrhosis cohort (N = 99).

Echocardiographic Parameters in Cirrhosis Cohort			
Echocardiographic Parameters	Results	Echocardiographic Parameters	Results
LA (mm)	39.15 (±6.21)	LVOT VTI (cm)	24.32 (±4.92)
LAV (mL)	79.77 (±26.79)	LV GLS (%)	20.52 (±2.93)
LAVi (mL/m^2^)	40.62 (±11.83)	SV (mL)	65.75 (±24.02)
E (cm/s)	75.04 (±18.41)	SVi (mL/m^2^)	30.91 (13.32–72.45)
A (cm/s)	68.06 (±18.69)	CO (L/min)	4.53 (±1.9)
DT (ms)	188.93 (±45.2)	CI (L/min/m^2^)	2.31 (±0.91)
TDI Septal e’ (cm/s)	8.7 (±2.17)	RA (mm)	38.56 (±5.41)
E/e’	8.97 (±2.53)	RAA(cm^2^)	16.9 (3.75)
E/A	1.19 (±0.49)	RV (mm)	34.39 (±4.87)
LASr (%)	32.93 (±8.57)	RVEDA (cm^2^)	19.15 (±5.28)
LAScd (%)	17.14 (±7.06)	RVESA (cm^2^)	9.68 (±2.78)
LASct (%)	15.8 (±5.22)	RV FAC (%)	49.27 (±6.79)
LV (mm)	48.37 (±5.66)	TDI RV S (cm/s)	14 (10–21)
LVi (mm/m^2^)	25.03 (±3.14)	TAPSE (mm)	24.48 (±3.85)
LVEDV (mL)	105.65 (±38.8)	RV/RA gradient (mmHg)	23.12 (±4.93)
LVEDVi (mL/m^2^)	53.91 (±18.15)	sPAP (mmHg)	28.25 (±5.12)
LVEF	62.53 (±6.04)	TAPSE/sPAP (mm/mmHg)	0.89 (±0.2)

**Table 3:** Abnormal echocardiographic and ECG findings in studied cirrhosis population (N = 99).

Abnormal Echocardiographic and ECG Findings in Cirrhosis Population (N = 99)			
Parameter	Nr. of Patients (%)	Parameter	Nr. of Patients (%)
LV hypertrophy	37 (37.4%)	LV GLS ≤ 18%	18 (18.2%)
LAVi ≥ 35 mL/m^2^	69 (69.7%)	LVEF ≤ 50%	2 (2.1%)
LAVi ≥ 45 mL/m^2^	35 (35.4%)	sPAP ≥ 35 mmHg	14(14.1%)
E/A ≤ 0.8	26 (26.2%)	Diastolic dysfunction (ASE 2016 criteria)	47 (47.5%)
DT > 200 ms	34 (34.3%)	Grade 1	26
DT ≤ 150 ms	22 (22.2%)	Grade 2	3
E/e’ ≥ 15	3 (3%)	Grade 3	4
E/e’ ≥ 10	35 (35.4%)	Indeterminate	14
TDI septal e’ ≤ 7 cm/s	30 (30.3%)	Long QTc (≥440 for males or ≥460 for females)	36 (36.4%)

**Table 4:** Prevalence of cirrhotic cardiomyopathy according to the utilized definition.

Prevalence of CCM According to the Utilized Definition		
	2005 CCM Criteria	2019 CCM Criteria
Systolic dysfunction		
Yes	10 (10.1%)	19 (19.2%)
No	89 (89.9%)	80 (80.8%)
Diastolic dysfunction		
Yes	56 (56.6%)	6 (6.1%)
No	43 (43.4%)	79 (79.8)
Indeterminate	0	14 (14.1%)
Cirrhotic cardiomyopathy		
Yes	60 (60.6%)	19 (19.2%)
No	39 (39.4%)	67 (67.7%)
Indeterminate	0	13 (13.1%)

**Table 5:** Correlations between liver disease severity (MELD Na) and echocardiographic parameters.

Correlations Between Liver Disease Severity (MELD Na) and Echocardiographic Parameters		
	Correlation Coefficient (Pearson)	Sig.
RVEDA (cm^2^)	0.259	0.01
RAA (cm^2^)	0.240	0.017
TAPSE (mm)	0.290	0.004
LAVi (mL/m^2^)	0.252	0.012
LASct (%)	0.229	0.023
LASr (%)	0.280	0.005
LV GLS (%)	0.289	0.004
CO (L/min)	0.403	<0.001
CI (L/min/m^2^)	0.380	<0.001
SV (mL)	0.384	<0.001

**Table 6:** Differences in echocardiographic parameters in patients with MELD Na score > 15.

Differences in Echocardiographic Parameters in Patients with MELD Na Score > 15							
Parameter	Yes (n = 42)	No (n = 57)	Sig.	Parameter	Yes (n = 42)	No (n = 57)	Sig.
E (cm/s)	81.1 (±18.4)	70.5 (±16.3)	0.004	SV (mL)	75 (±27.5)	58.8 (±18.4)	0.001
LV GLS (%)	21.5 (±3.2)	19.7 (±2.5)	0.002	SVi (mL/m^2^)	34.9 (20–72.5)	30.5 (13–57)	0.001
LVOT VTI (cm/s)	26.3 (±5.1)	22.8 (±4.2)	<0.001	CO (L/min)	5.3 (±2)	3.9 (±1.6)	<0.001
LVTDV (mL)	119.3 (±46.9)	95.6 (±27.9)	0.002	CI (L/min/m^2^)	2.7 (±0.96)	2 (±0.75)	<0.001
LVTDVi (mL/m^2^)	59.9 (±21.6)	49.5 (±13.7)	0.005	sPAP (mmHg)	30.1 (±5.2)	26.8 (±4.5)	0.001
LAVi (mL/m^2^)	43.9 (±13.6)	38.1 (±9.8)	0.01	TDI RV S (cm/s)	14 (10–21)	13 (10–17)	<0.001
LA (mm)	41.2 (±6.8)	37.7 (±5.2)	0.005	TAPSE (mm)	25.7 (±4.4)	23.6 (±3.1)	0.007
LV (mm)	50.3 (±5.7)	47.04 (±5.2)	0.003	RV/RA grd. (mmHg)	24.9 (±4.7)	21.8 (±4.6)	0.001
LASr (%)	36.1 (±8.9)	30.6 (±7.5)	0.001				

**Table 7:** Parameters that correlated with survival time (days) in deceased patients.

Parameters That Correlated with Survival Time (Days) in Cirrhotic Patients						
	Correlation Coefficient	Sig.	Parameter/Time (Days)	Yes	No	Sig.
MELD	−0.489	0.007	Pleural effusion	85.9 (±65.5)	247.1 (±159.9)	0.007
MELD Na	−0.553	0.002	LASct < 16	291.2 (±181.5)	139.6 (±107.1)	0.008
TAPSE/sPAP (mm/mmHg)	0.459	0.01	LAVi ≥ 45 mL/m^2^ (days)	115.4 (±70.2)	240.1 (±172.4)	0.038
RV/RA gradient (mmHg)	−0.391	0.036	MELD ≥ 15 (days)	137.1 (±105.7)	258.9 (±175.7)	0.037
sPAP (mmHg)	−0.399	0.032	Meld Na ≥ 15 (days)	139.5 (±112.4)	295.4 (±179)	0.006
LASct	−0.373	0.046				

**Table 8:** Multivariable Cox proportional hazards survival analysis.

Covariate	Sig.	HR	95% CI	
			Lower	Upper
Meld Na	0.006	1.094	1.026	1.165
LAVi	0.031	1.045	1.004	1.087
LAS ct	0.682	0.985	0.916	1.059

**Table 9:** Binomial logistic regression for composite end-point (death or LT).

Independent Variables	Sig.	OR	95% CI	
			Lower	Upper
Meld Na	0.047	1.076	1.001	1.157
Pleural effusion (1)	0.018	4.683	1.306	16.826
Diastolic Dysfunction (1)	0.009	4.876	1.481	16.058

## Data Availability

Data is available upon request by e-mail to corresponding author.

## Abbreviations

The following abbreviations are used in this manuscript:

ESLD	End-stage liver disease
RAAS	Renin–angiotensin–aldosterone system
SNS	Sympathetic nervous system
LT	Liver transplant
HF	Heart failure
CCM	Cirrhotic cardiomyopathy
HCC	Hepatocarcinoma
MELD	Model for end-stage liver disease
CAD LT	Coronary artery disease in liver transplantation
CW	Continuous wave
PW	Pulse wave
LA	Left atrium
LAVi	Left atrial volume index
LAV	Left atrial volume
LV	Left ventricle
LVEDV	Left ventricle end diastolic volume
LVEDVi	Left ventricle end diastolic volume index
LVESV	Left ventricle end systolic volume
LVEF	Left ventricle ejection fraction
LV GLS	Left ventricle global longitudinal strain
RV	Right ventricle
RVEDA	Right ventricle end diastolic area
RVESA	Right ventricle end systolic area
FAC	Fractional area change
RA	Right atrium
RAA	Right atrium area
IVC	Inferior vena cava
TAPSE	Tricuspid annulus plane systolic excursion
CO	Cardiac output
CI	Cardiac index
SV	Stroke volume
SVi	Stroke volume index
SVR	Systemic vascular resistances
CRP	C reactive protein
HFpEF	Heart failure with preserved ejection fraction
HFrEF	Heart failure with reduced ejection fraction
CAD	Coronary artery disease
LAS	Left atrial strain
LASr	Left atrial reservoir strain
LASct	Left atrial contraction strain
LAScd	Left atrial conduit strain
BSA	Body surface area
BMI	Body mass index
TIPS	Transjugular intrahepatic portosystemic shunt
ASE	American society of echocardiography
VHD	Viral hepatitis D
VHB	Viral hepatitis B
VHC	Viral hepatitis C
sPAP	Systolic pulmonary artery pressure
